# Volcano electrical tomography unveils edifice collapse hazard linked to hydrothermal system structure and dynamics

**DOI:** 10.1038/srep29899

**Published:** 2016-07-26

**Authors:** Marina Rosas-Carbajal, Jean-Christophe Komorowski, Florence Nicollin, Dominique Gibert

**Affiliations:** 1 Institut de Physique du Globe de Paris, Sorbonne Paris Cité, CNRS UMR-7154, Université Paris Diderot, Paris CEDEX 05, France; 2 Géosciences Rennes, Université Rennes 1, 35000 Rennes, France

## Abstract

Catastrophic collapses of the flanks of stratovolcanoes constitute a major hazard threatening numerous lives in many countries. Although many such collapses occurred following the ascent of magma to the surface, many are not associated with magmatic reawakening but are triggered by a combination of forcing agents such as pore-fluid pressurization and/or mechanical weakening of the volcanic edifice often located above a low-strength detachment plane. The volume of altered rock available for collapse, the dynamics of the hydrothermal fluid reservoir and the geometry of incipient collapse failure planes are key parameters for edifice stability analysis and modelling that remain essentially hidden to current volcano monitoring techniques. Here we derive a high-resolution, three-dimensional electrical conductivity model of the La Soufrière de Guadeloupe volcano from extensive electrical tomography data. We identify several highly conductive regions in the lava dome that are associated to fluid saturated host-rock and preferential flow of highly acid hot fluids within the dome. We interpret this model together with the existing wealth of geological and geochemical data on the volcano to demonstrate the influence of the hydrothermal system dynamics on the hazards associated to collapse-prone altered volcanic edifices.

Flank instability is a common process in the evolution of volcanic edifices of all types and geodynamic settings. Massive partial flank collapse of stratovolcanoes have occurred at about 200 volcanoes in the last 10,000 years and caused about 20,000 fatalities^1^,^2^,^3^. Roughly 52% of historical collapses since 1500 AD are triggered and linked to magmatic activity, however about 22% are associated with hydrothermal eruptions^1^,^2^,^3^. Detecting and assessing unrest that could herald the onset of non-magmatic collapse of a hydrothermally-altered and cored volcano constitutes a significant challenge with current volcano monitoring techniques for Earth scientists and civil protection authorities.

In active volcanoes, hydrothermal systems develop in the host-rock where heat is transferred from the magmatic deeper source to the surface via free convection that involves magmatic fluids (gas or liquid) and meteoric recharge fluids. The behavior and dynamics of the magmatic system can modify the geophysical and geochemical signature of processes associated with the hydrothermal system. Hydrothermal fluids are enriched in ions resulting from the physico-chemical and thermal exchanges between meteoric water, magmatic gases, heat, and the host-rock. Hot hydrothermal fluids (<400 °C) are enriched in dissolved, or coexisting with, magmatic gases (for example H_2_S, SO_2_, HCl, and HF) such that they are particularly acid (pH < 0). These fluids promote intense leaching and alteration of the host-rock (high dissolution rates^4^,^5^ on the order of 1–2 kg.s^−1^) that can lead to mechanical weakening of the edifice^4^,^5^,^6^,^7^ and considerably reduce the frictional force needed to achieve displacement in a fault^8^.

Numerical modeling has shown that heating of hydrothermal fluids saturating porous host-rock can drastically increase fluid pressures in localized zones of the volcanic edifice^9^ and trigger partial edifice collapse and laterally directed explosions^2^,^10^. Indeed, catastrophic volcano collapses are often associated with laterally-directed explosive depressurization of hydrothermal fluids that lead to the emplacement of lethal high-energy dilute turbulent mixtures of rock fragments and hot gases (i.e. pyroclastic density currents). Recently, hydrothermal laterally-directed explosions on a smaller scale caused 63 fatalities at Ontake volcano in 2014^11^ and could have caused many fatalities at Tongariro in 2012^10^,^12^. Non-magmatic hydrothermal blasts are as mobile as their magmatic counterparts^12^,^13^. The often unpredictable sudden onset of these hydrothermal volcanic blasts poses significant threats to population and infrastructure exposed within less than 5 km from the source areas on many high hazard volcanoes^14^ such as at La Soufrière de Guadeloupe (see Supplementary Information).

Knowledge of the subsurface structure and dynamics of the volcanic hydrothermal system and other structures that contribute to edifice instability such as faults, detachment planes and areas of mechanically weak rock is thus crucial for risk assessment in volcanic systems^9^,^15^,^16^. It also constitutes key information to determine their role in eruptive processes. These structures can be complex and vary markedly among volcanic systems and within zones of the same volcano, therefore a priori knowledge about them is rare^17^. Imaging of the hydrothermal system, together with geophysical and geochemical monitoring data, helps to construct a functional dynamic model of the volcanic edifice linking observables, hidden states of the hydrothermal-magmatic system, and possible eruptive outcomes^18^. This knowledge contributes to reduce epistemic uncertainty in modelling constraints (e.g. volume, overpressure, path) for hazard and risk-oriented simulations of partial edifice collapse (for example^19^,^20^), associated laterally-directed explosions and potentially tsunamigenic debris avalanches at volcanoes in populated areas (for example^21^).

Electromagnetic geophysical methods, such as electrical resistivity tomography, are sensitive to electric conductivity anomalies associated with fluid-saturated rocks and have been widely used to image fluid presence and migration^22^. Natural liquid fluids have electrical conductivities higher than that of the rock matrix, and relatively small amount of liquids can increase bulk rock conductivity by several orders of magnitude, even more so if they form an interconnected network^23^. Electrical conductivity in the volcano edifice will thus be larger where liquid saturated rocks are more porous and permeable, that is, where fractures, faults and altered rock are found. The electrical conductivity of the rock will also be larger for hotter and more saline liquids^24^. Clay minerals in hydrothermally altered rocks also contribute to a higher electrical conductivity^25^. Measurements of electrical conductivity can therefore be used to constrain the volume of fluid-saturated rocks in the subsurface, the mechanical properties of the volcano, and the character of the saturating fluids. Nearly all previous electrical resistivity tomography surveys on volcanoes were limited to either exclusively two-dimensional models (for example^26^,^27^) or three-dimensional models based on two-dimensional acquisitions (for example^28^,^29^).

The La Soufrière de Guadeloupe volcano is an archetype of hazardous andesitic volcanoes that occur in many subduction zones such as the Lesser Antilles Volcanic Arc (Fig. 1a). It is characterized by a great diversity of eruptive styles^30^,^31^ (see Supplementary Information). Its lava dome (Fig. 1b; Supplementary Fig. S1) was formed during the last major magmatic eruption in 1530 AD^32^. Since 1635 AD, six non-magmatic phreatic or hydrothermal eruptions have occurred^30^ associated with significant exurgence of hot acid hydrothermal fluids stored in reservoirs within the edifice (see Supplementary Fig. S2). There is currently significant uncertainty whether the slowly increasing unrest recorded since 1992^30^,^33^,^34^ will lead to hazardous eruptive activity linked to hydrothermal and/or magmatic processes for nearby communities. Extensive interdisciplinary research and monitoring programs spanning geological, geophysical, and geochemical approaches and techniques (see for example^33^,^34^,^35^,^36^,^37^,^38^,^39^) have focused on La Soufrière de Guadeloupe volcano since the last eruption in 1976–1977^18^,^30^,^34^,^40^.

In this paper, we study the structure and dynamics of the hydrothermal system of the La Soufrière de Guadeloupe lava dome. We invert extensive electrical tomography data collected throughout the dome to obtain a three-dimensional model of its electrical conductivity distribution. We then interpret this model integrating geological and geochemical data to build a conceptual model of the hydrothermal system’s structure and dynamics. This model shows that a genuine hazard of partial edifice collapse exists for the southern flank of the dome. We provide estimates for the potential volume of material involved for different scenarios and compare these to past edifice collapses studied at La Soufrière de Guadeloupe^30^,^31^,^41^.

## Results

**Figure 1:**
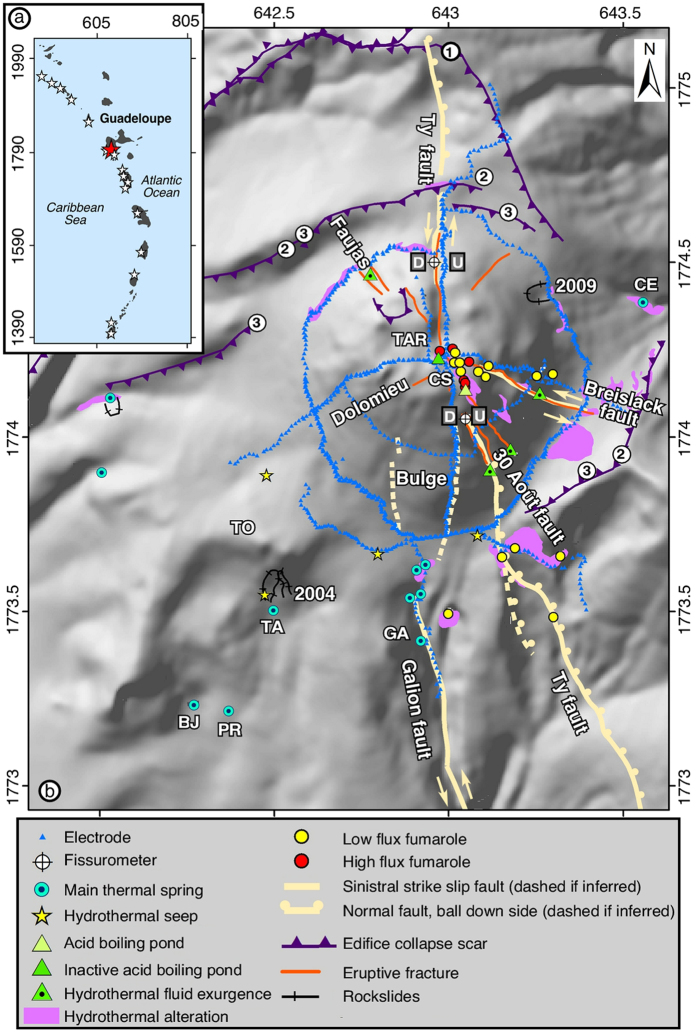
Location and main structures of the La Soufrière de Guadeloupe lava dome and hydrothermal system. (**a**) Holocene volcanoes (stars) in the West Indies active in the last 10,000 years with La Soufrière de Guadeloupe as red star (details in Supplementary Information). (**b**) The circle described by the electrodes follows the base of the lava dome. Numerous surface manifestations of hydrothermal activity are present in the dome and surroundings with the main high flux fumaroles of Tarissan (TAR) and Cratère Sud (CS) and the thermal springs of Galion (GA), Carbet Echelle (CE), Tarade (TA), Pas du Roy (PR), and Bains Jaunes (BJ). Past edifice collapse structures are labelled 1 (3060 years BP event, BP, i.e. before 1950 AD); 2 (3 events younger than 2900 years BP) and 3 (1530 AD event). TO: Tarade Toreva collapse megablock. The sense of movement of the Ty fault derived from fissurometer data is shown as U (up) and D (down). This map was generated using the Esri ArcMapI 10.1 software (http:www.esri.com).

We conducted electrical resistivity tomography experiments with electrode transects extending through the La Soufrière lava dome and around it (see electrodes in Fig. 1b). More than 23,000 data points were acquired with two- and three-dimensional protocols, including several pairs of injection electrodes located on opposite sides of the volcano to better resolve the innermost regions of the lava dome^39^. Some of the measurements were performed with one of the current electrodes immersed in the Tarissan boiling acid pond (Fig. 1b). The pond is extremely conductive (∼25 S.m^−1^ at ambient temperature)^34^ and acts as a large electrode extending through the inner conductive structure of the volcano effectively conducting the current inside the volcano. This disposition provides better constraints on the volcano’s internal structure.

**Figure 2:**
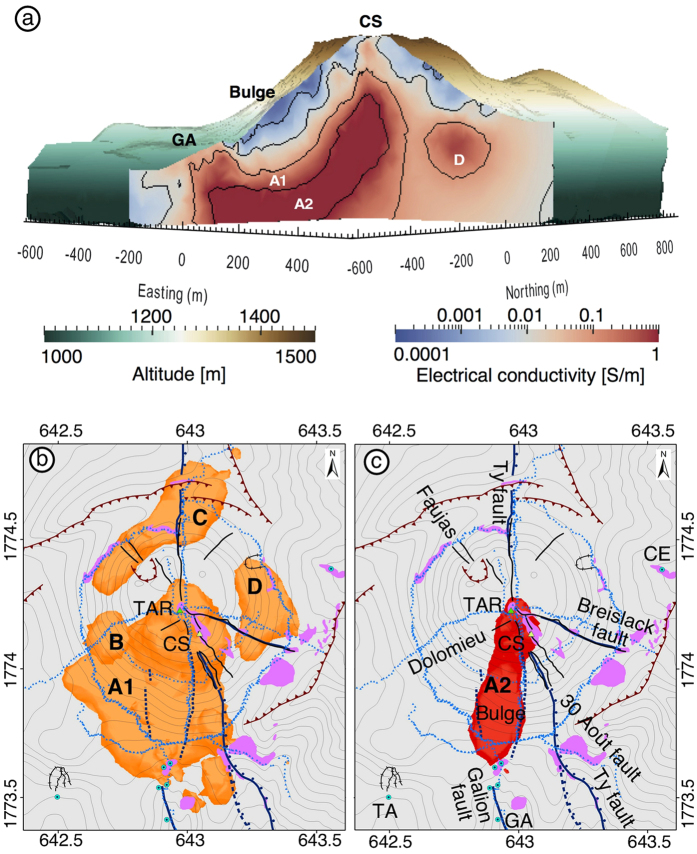
Electrical conductivity inverse solution of the La Soufrière de Guadeloupe lava dome. (**a**) South-north and east-west slices of electrical conductivity seen from the south-east, depicted in a blue-to-red colour scale where red colour coding represents more conductive material and thus larger amount of liquids and higher temperatures. Top surface shows the altitude with a green-to-brown scale. (**b**,**c**) Regions with electrical conductivity larger than 0.1 (**b**) and 1 (**c**) S.m^−1^, viewed from above, with the structures presented in Fig. 1 superposed. The “A2” anomaly in c) is contained inside the “A1” anomaly in b) (see iso-conductivity line contours in a). See Fig. 1b for legend and line symbols. The maps in Fig. 2b,c) were generated using the Esri ArcMapI 10.1 software (http:www.esri.com).

We calculated the three-dimensional electrical conductivity distribution of the La Soufrière de Guadeloupe lava dome (Fig. 2a) applying a regularized inversion algorithm to the data^29^ that solved for the conductivity of more than 1,000,000 mesh cells. We used a digital elevation model of 15 m resolution to model the topography in the central part of the modelling and inversion mesh (see Supplementary Fig. S6). The depth limit of 550 m below the summit of the conductivity model shown in Fig. 2a corresponds to the sensitivity limit of the data to changes in the conductivity model (Supplementary Fig. S10).

Several conductive regions (conductivity larger than 0.1 S.m^−1^, Fig. 2b) are present in the lava dome. Sensitivity tests were performed to assure that all of these regions are required by the model to fit the data (see Supplementary Information). The largest is located in the volcano’s southern flank (Fig. 2a,b, label “A1”) and contains a sub-region with conductivity values larger than 1 S.m^−1^ (Fig. 2c, label “A2”). It presents a concave upward listric structure (Supplementary Fig. S11) and spreads from the summit to the south, where it is structurally controlled by the sinistral strike-slip 30 Août^42^,^43^ and Ty faults^44^ and by the prolongation of the Dolomieu fracture from the summit to the base of the lava dome, prevailing in surface area through almost a quarter of the lava dome’s flanks (Fig. 2b). Above the central part of the anomaly there is a resistive bulge of dense rock (Figs 1b and 2a). The model also indicates smaller conductive anomalies beneath the west, north and east flanks of the lava dome (labels “B”, “C” and “D” in Fig. 2b) that do not reach conductivity values of 1 S.m^−1^.

The extremely conductive (conductivity larger than 1 S.m^−1^) region A2 is oriented south to slightly south-west in the direct prolongation of the sinistral strike-slip Galion fault plane^45^ (Fig. 2c). The boundaries of A2 correlate with structurally controlled gullies that have negative self-potential anomalies^38^. These anomalies are associated to the infiltration of meteoric water through a structural lineament in the prolongation of the Galion fault (Fig. 1b).

**Figure 3:**
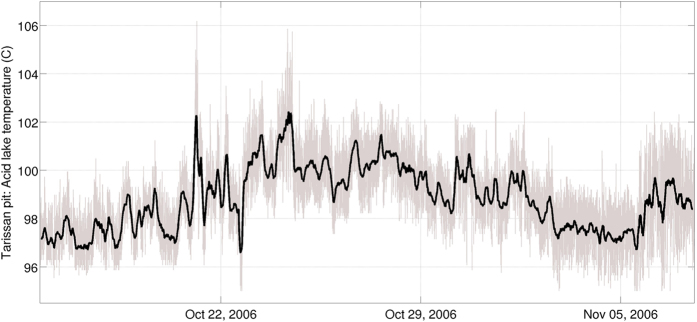
Temperature variations at the Tarissan acid pond. Measurements were taken between October 15 and November 07, 2006. The grey line represents measurements done every minute and the black line shows a smoothed version (average over 16 hours) of these variations. Sudden temperature increases in the acid pond are frequent, with periods of increasing temperature of between 10 and 20 hours. These temperature increases correspond to the arrival of new, hotter magmatic fluids to the pond.

Region A2 also presents a listric structure (sub-vertical below the summit to horizontal below the Galion thermal springs, Fig. 2a). The upper part of A2 is connected to the summit’s most active region of degassing fumaroles (Cratère Sud) and to the acid pond in the Tarissan pit at the intersection of the 30 Août and Breislack faults (Figure 1b). Figure 2a shows a conduit of ∼0.05 S.m^−1^, which extends from A2 to the surface. This corresponds to the highly altered rocks observed in the Cratère Sud region (Supplementary Fig. S3). Conversely, the Tarissan pit is surrounded by otherwise unaltered solid rock (Supplementary Fig. S3). Hot, acid liquids (97 °C, −1 < pH < 0) are present about 80 m below the summit in this pit where the upper limits of the A2 anomaly are observed. Alteration and dissolution of the andesite host-rock leads to an enrichment of these liquids in chlorine (maximum contents of 10 wt.% Cl^-^) and ions (Ca^++^ = 3500 ppm, Mg^++^ and Na^+^ = 1200 ppm)^34^ that contributes to increase their electrical conductivity. Sudden temperature variations are frequent in the pond (Fig. 3). Abrupt temperature decreases likely correspond to heavy rain episodes whereas sudden increases strongly suggest the arrival of new pulses of hotter magmatic fluids.

## Discussion

Fluids sampled in fumaroles and thermal springs at La Soufrière de Guadeloupe vary from a pure magmatic gas end-member, rich in sulphur and chlorine, to a pure meteoric water end-member^34^. As magmatic gases rise to the summit they cool and condense to form magmatic liquid fluids. They mix with meteoric water in shallow aquifers and form a gravitationally-controlled groundwater flow. Five out of six of its historical non-magmatic hydrothermal eruptions have triggered the exurgence of pressurized warm to hot acid fluid from eruptive fractures^30^ (see Supplementary Information and Supplementary Fig. S2). This provides unequivocal evidence of the presence, within La Soufrière de Guadeloupe dome, of numerous possibly interconnected reservoirs of aggressive acid hydrothermal fluid.

**Figure 4:**
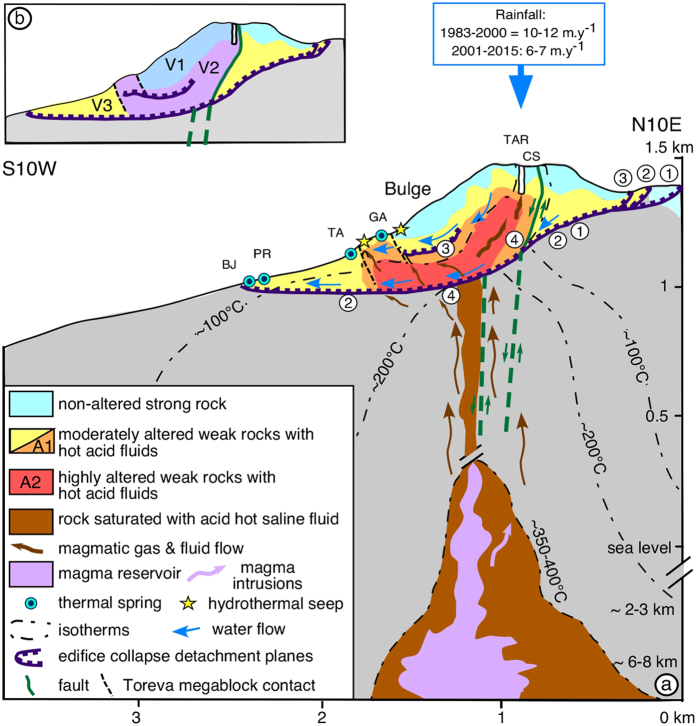
Links between hydrothermal system dynamics and edifice instability for La Soufrière de Guadeloupe. (**a**) Degassing of the magma reservoir and shallow-depth magma intrusions produce hot acid magmatic fluids that weaken the core of the volcanic edifice. The argilized detachment plane favours magmatic fluid flow parallel to it, while flow is almost vertical from the detachment plane to the summit. The listric disposition of the highly altered, weak, fluid-saturated zones and the low basal friction of detachment planes imply a partial edifice collapse hazard. The conceptual model of the structure and dynamics of the hydrothermal system at La Soufrière de Guadeloupe is inferred from the three-dimensional electrical tomography model and collapse scars observed on the surface (see Fig. 1 and references on numbering of detachement planes therein) combined with geological data on past eruptive activity, faults, the geochemistry of thermal springs, fluid flow tracer tests, and fissurometer monitoring data. Number 4 corresponds to the detachment plane of a future collapse scenario. The Galion, 30 Août and Ty sinistral strike-slip faults are sub-parallel to the cross-section and are shown with a green line. Dashed green lines refer to inferred faults below the dome. (**b**) Sub-division of regions based on the iso-conductivity surfaces shown in Fig. 2 used to estimate the order of magnitude of potential collapse volumes.

There is strong evidence that the dome largely consists of hydrothermally altered rocks^30^,^31^,^46^,^47^. Hydrothermal alteration has been shown to cause order-of-magnitude scale decreases in the mechanical strength of rocks by reducing friction, rock strength, and cohesion^7^. Surface outcrops of altered rock are abundant (Fig. 1b). Debris avalanche deposits from past collapses of the different edifices over the last 9150 years and ash products of the explosive hydrothermal eruption of 1976–1977 are constituted dominantly by altered material containing a pervasive assemblage of clay minerals, silica polymorphs, sulphates, and sulfides^46^,^47^. Such an assemblage is typical of acid-sulphate argilic alteration by fluids percolating through the upper part (<1 km depth) of the edifice within the lower temperature region (<150°) of a magmatic-hydrothermal system (Fig. 4a)^5^,^46^. The Galion and Tarade thermal springs (Fig. 1b) are enriched in dissolved silica^48^. The small 2009 landslide contained abundant highly altered clay-rich material (Fig. 1b and Supplementary Fig. S5) resulting from efficient alteration within the dome.

The chlorine-rich signature of high-flux degassing at La Soufrière de Guadeloupe is diagnostic of the injection of new small-volume batches of chlorine and water-rich silicic magma that stall at a few kilometers below the summit^34^. The acid ponds enriched in magmatic chlorine that developed in the Cratère Sud (See Supplementary Information and Supplementary Fig. S4) and Tarissan pit since 1998 constitute proxies to the magmatic fluid contained in the volcano’s hydrothermal reservoirs. Moreover, the proximity of the Tarissan acid pond to the A2 anomaly of extreme conductivity suggests that its fluid is similar in composition to that of the Tarissan pond. We expect the fluid temperature in the anomaly to be higher, because in the pond magmatic fluids are mixed with meteoric fluids (rainfall average is ∼6–7 m.y^−1 ^)^34^. The A2 anomaly thus represents the major path for chlorine-rich acid fluids (gas and liquid) ascending from the deep magmatic reservoir (Fig. 4a). Prolonged hydrothermal alteration characterized by high dissolution rates^4^,^5^ caused by these fluids leads to significant mechanical weakening of the host-rock in this region^7^.

The La Soufrière de Guadeloupe dome is affected by two main faults (Fig. 1b): the sinistral strike-slip Galion fault (strike N170E)^45^, and the high angle normal Ty fault (strike N175E) that dips to the east^44^. On the southern flank, the Ty fault connects with the 30 Août fault (strike N150E) that formed during the 1976 eruption 30,47. The Galion and 30 Août faults join the sinistral strike-slip Breislack fault (strike N120E)^42^,^43^ in the Tarissan area and propagate north as the Ty fault (N0E strike). Forty years of fissurometry data on the southern and northern flanks of the dome (OVSG-IPGP, personal communication; Fig. 1b) show a present-day sinistral strike-slip movement with a downward component to the west for the active Ty fault. The southwest sectors of the dome between the Galion, 30 Août, and Breislack faults are experiencing a complex clockwise rotational and downward gravitational spreading movement to the southwest in the direction of the greatest unbutressed slope.

The structure of the conductivity model suggests a connection between the summit and the southern thermal springs through the A2 and A1 regions (Fig. 2) controlled by the merging of the Galion and the La Ty/30 Août faults (Fig. 4a). Indeed, geochemical analyses have shown a genetic link^34^,^49^ between the chemical composition of fumaroles, acid ponds, and the most proximal thermal springs such as Galion, Tarade, Pas du Roy and Bains Jaunes (see Figs 1b and 4a). The acid ponds have normalized Mg-Ca-Na compositions similar to the thermal springs but are highly enriched in chlorine with negligible SO_4_^–^ and HCO_3_^–^ concentrations^34^. The Galion spring showed, from 2001 to 2010, a series of Cl anomalies that were non periodic and of increasing intensity with time^34^. These anomalies are not correlated to spring temperature increase, rainfall or seismic activity however, they are clearly correlated, although with a significant time delay, to the onset in 1998 of chlorine degassing in summit fumaroles^34^. Moreover, spring waters are enriched in Zn and in the heavier Zn isotopes (i.e. ^66^Zn) compared to andesite host-rock^50^, with the Tarade and Galion thermal springs being the most enriched. This is the result of hydrothermal alteration of the host-rock taking place within the dome^50^ (Fig. 1b).

The incorporation of this geological and geochemical data to the three-dimensional conductivity model of the lava dome allows us to infer the dynamics of the hydrothermal fluid flow in the dome. Magmatic fluids preferentially follow an essentially vertical path to the summit through the 1530 AD eruptive conduit (Fig. 4a). Fluids outflowing at the Galion springs along the Galion fault are a combination of magmatic fluids arriving from the major magmatic fluid conduit (A2 located at ∼150 m below the surface in this region), and magmatic fluids that arrive at the summit and then mix with down-flowing meteoric water (Fig. 4a). This downhill flow takes place in shallow parts of the edifice along the Galion and the Ty / 30 Août faults and associated fractures (Fig. 4a), as inferred from self-potential anomalies^38^,^51^. The flow is also controlled by the detachment plane of the most recent collapse structure on top of which the lava dome was built in 1530 AD^30^ (label 3 in Figs 1b and 4a).

The prolongation to the south of the major magmatic fluid path most likely occurs along older and larger collapse listric structures (labels 1 and 2 in Figs 1b and 4a), that are observed at the surface north, northwest, and east of the dome. The depth of the associated detachment planes is inferred from several lines of evidence. Thermal springs are located to the south-southwest exclusively along a common elevation contour inverval and define an inclined plane from 1100 m to 975 m of elevation. No thermal springs occur to the southwest and south of the volcano below 975 m. Two-dimensional electrical tomography data^38^ revealed a high conductivity unit at a depth of 80 m below the bulge on the south flank. Analysis of core sections from a 97 m well in that region show that this unit consists of clay-rich and hydrothermally altered deposits that lie below highly shattered, fractured, and altered lava^52^. Field observations of a major transversal ridge above the Tarade spring (Fig. 1b; Fig. 4a)^30^,^31^ show that it consists of the same lava unit now steeply tilted to the North and resting more than 50 m above its position in the well nearby. The lower portion of the Tarade ridge consists of a thick lava breccia with a clay-rich matrix saturated with hydrothermal fluids (seep on Fig. 1b). This ridge is interpreted^30^,^31^ as a Toreva edifice collapse megablock resting above a matrix of debris avalanche material that forms the high conductivity basal detachment plane in this area. The compressional gravitational spreading of the southwest sector of the dome against the buttressing Tarade megablock is likely responsible for the formation of the observed bulge (Figs 1b and 4a) as well as the rise of hydrothermal fluids in the Tarade area. Finally, in 1994 a chemical tracer (KI) injected in the Tarissan pit was recovered to its 25% by weight at a distance of 600 m from the axis of the dome (Bains Jaunes spring, Fig. 1b), with 60% of the tracer recovered within 3.5 months from the onset of the experiment. This provided evidence of preferential fluid flow towards the southwest^35^ along a major structurally controlled pathway. At the time of the experiment the tracer flow was unimpeded by any acid pond or gas flux^30^,^49^.

The less conductive sub-region (lower than 1 S.m^−1^) of anomaly A1 most likely consists of liquid saturated rock that is continuously altered by the aggressive hot acid fluids migrating from the major fluid conduit. Geochemical analysis of thermal springs located near the base of the dome (Galion, Tarade, Figs 1b and 4a) near A1 support this hypothesis in as much as these springs are characterized by ions of magmatic origin such as HCO_3_^–^, HCl, and SO_4_^–^ and ions produced by hydrothermal alteration such as Mg^++^ and Ca^++^, and heavy zinc isotopes such as ^66^Zn^34^,^50^. We suggest that the lower temperature (between 100° and 30°) arising from the mixture of meteoric and magmatic fluids is the main cause of conductivity values lower than in the A2 region (Fig. 4a), whereas a strong hydrothermal alteration is also persistent in the less conductive sub-region of A1. Indeed, in the Galion spring region where hydrothermally altered rocks and thermal springs are observed at the surface, the A1 conductor reaches the surface (Fig. 2 and Supplementary Fig. S11). The 2009 landslide revealed abundant altered material above the D conductor where the model indicates a weaker conductivity. Thus, hydrothermally altered rock can be present even in regions where conductivity does not reach values as high as 0.1 S.m^−1^. The lower conductivity is probably due to a reduced liquid saturation since this region is near to the surface.

The south and southwest flanks of the lava dome are laying above a low-strength region that is continuously mechanically weakened by intense hydrothermal alteration. The listric structure of A1 indicates that this large hydrothermal reservoir is confined between two low-strength detachment planes (Fig. 4a). The high fluid flux to the summit fumaroles and the sudden onset of sustained chlorine degassing observed at the summit since 1997–1998 coupled with the geochemical evolution of the Galion springs^33^,^34^,^49^ indicate that progressive self-sealing by prolonged argilic alteration promotes the flow of magmatic fluids along discrete structures such as faults and fractures (Fig. 4a) and then parallel to the basal detachment plane. Indeed, a significant time delay is observed between the occurrence of the maximum HCl content in summit fumaroles in 2000–2001 and the maximum concentration of HCl in the Galion spring in 2009^34^. This sealing limits the process of scrubbing of acid gases such as chlorine by the shallow meteoric fluid downflow and favours fluid saturation. This further promotes intense hydrothermal alteration in the A1 region and mechanical weakening of the dome’s core above the detachment plane, in an area that has experienced recurrent pervasive alteration after each past collapse event^30^,^31^,^41^.

The smaller conductive regions labelled “B”, “C”, and “D” probably consist of liquid-saturated, hydrothermally altered material remanent from past hydrothermal activity in the dome. No surface hydrothermal activity is presently observed in the vicinity of these regions. Figure 2b shows evidence of past hydrothermal alteration at the surface above structures C and D. Anomaly C is structurally correlated with the dome’s base and probably corresponds to the floor of the argilized detachment plane of the 1530 AD edifice collapse^30^ (Figs 1b and 4a). A prolongation of the conductor C towards the surface (Supplementary Fig. S11) is correlated to the Faujas fracture, which experienced hydrothermal fluid exurgence in 1836–37 (Supplementary Fig. S2). The D anomaly is located below the region where the 2009 rockslide revealed altered material (Fig. 1b and Supplementary Fig. S5). Given its proximity to the large conductor A1, we expect conductors B and D to be connected to this large hydrothermal reservoir, although through reduced paths that are not resolved by the electrical tomography. Indeed, low flux fumaroles are located above the D anomaly (Fig. 1b).

The listric and high-angle disposition of the A1 region, its mechanical weakening due to the alteration caused by hot acid liquids, and its confinement between the argilized detachment planes indicate a mechanical proneness to partial edifice collapse. Pore-fluid pressure propagation caused by a remote magma intrusion or from the magmatic fluids reaching the shallow hydrothermal system could trigger the collapse^9^. At La Soufrière de Guadeloupe, the 5 partial edifice collapses of the last 3,200 years, all associated with south-southwestward detachment planes (Figs 1b and 4a)^30^,^31^,^53^, emplaced debris avalanche deposits in that direction into the populated areas of Saint-Claude and Basse Terre and to the coastline, 9 km from the dome.

Based on the A1 anomaly and knowledge of the ancient collapse structures (Fig. 1b; Fig. 4a), we estimated the volume of material that could be involved in potential sector collapses of the lava dome (Fig. 4b). We calculate a minimum collapse volume of ∼35 × 10^6^ m^3^ when only considering the material above the upper surface of A1 (Fig. 2a and “V1” in Fig. 4b). This scenario would correspond to a collapse re-using the 1530 AD detachment plane (number 3 in Fig. 4b). The estimated volume is of the same order of magnitude as the estimates for the actual 1530 AD collapse volume (i.e. 80 +/− 40 × 10^6^ m^3^)^31^. We obtain a larger volume of 115 × 10^6^ m^3^ (“V1 + V2” in Fig. 4b) when we take into account the altered material (“V2”) comprised from the interpreted basal limit of A1 (reactivating the main detachment plane of the last 5 events, Fig. 4b) to the top of A1 as well as material in “V1” located above to the surface. These estimates are comparable to the volumes (80–350 × 10^6^ m^3^) estimated for the last 5 edifice collapses at La Soufrière de Guadaloupe^31^. A collapse involving this amount of material would probably develop along the detachment plane numbered 1 and 2 in the southern part of the profile in Fig. 4a. The provided volume estimates do not take into account the material comprised between the zone where the conductor ends and the location where the detachment plane intersects the surface (“V3” in Fig. 4b). Thus, simulations of the emplacement of potential future debris avalanches should comprise a larger volume than our estimates. Nevertheless, these estimates constitute valuable conservative approximations for the 2 detachment plane scenarios.

A partial edifice collapse hazard has long been suggested at La Soufrière de Guadeloupe given its history of collapses and supporting geological and geochemical data^19^,^30^,^31^. Our findings build on these data and a comprehensive structural analysis to provide unprecedented details of the dynamics of one of the most hazardous volcanic hydrothermal systems in the world^14^. These dynamics bear a strong influence on the particularities of each collapse event (location and geometry of collapsed structure, detachment plane, volume of material and fluids involved). By identifying the preferential zones of hydrothermal fluid circulation and the different structures that favor mechanical instability, we reduce the uncertainty linked to the quantification of risk for non-magmatic eruptions (lateral directed explosions and associated pyroclastic density currents, partial flank collapse, large reservoirs of hot acid fluid feeding aggressive contaminating mudflows) as well as the early phases of magmatic eruptions.

Our model constitutes effective prior information needed for reliable instability analysis, simulation of flank collapse and directed explosion dynamics as well as consequent risk mitigation. Numerous otherwise low unrest volcanoes in a “phreatic state”^14^ could cause high impact in populated areas even with meteoric, seismic, or hydrothermal forcing. Many volcanoes of this class present risks but have no or limited monitoring equipment^14^. Knowledge of their hydrothermal system dynamics and their structural controls is paramount to identify the preferential zones for monitoring and to constrain the range of possible eruptive scenarios to be considered in effective and timely risk mitigation.

## Methods

### Electrical resistivity tomography

Electrical resistivity tomography is a geophysical method for imaging the electrical conductivity (reciprocal to resistivity) structure of the subsurface. The spatial variation of conductivity in the field is determined using four-electrode measurements. Two injection electrodes create an electrical circuit, while the other two measure the potential difference (voltage). The measured transfer resistance is given by *R* = *V*/*I*, where *V* is the voltage and *I* is the injected current. Electrodes may be placed on the ground surface, bore-holes or extremely conductive bodies to create a large equipotential^54^. Inverse methods are usually applied to the data to find the subsurface conductivity structure that explains such measurements.

### Data sets

Data were collected in a series of field experiments between 2003 and 2011 resulting in more than 23000 measurements (See Supplementary Fig. S7). Table 1 shows the number of data collected in each field campaign. The large time span necessary to perform such amount of measurements raises the question of whether significant changes occurred in the volcano structure during the acquisition. To test this hypothesis several profiles were repeated at different campaigns. Differences in the data from the overlapping profiles are similar to differences found in profiles repeated during the same campaign slightly varying the electrode position (∼50 cm). We separately inverted the measurements repeated with the largest time difference (south-north profiles on the west side of the volcano of the December 2003 and July 2011 campaigns) to investigate possible coherent changes in the model. We performed independent inversions with the same homogeneous starting model and also a time-lapse inversion^55^ where the final model of the data set including the 2003 measurements was used as a starting and reference model for the inversion of the most recent data. No significant differences were found in the conductivity models with similar data fit in any of the cases. Thus, any changes in the volcano during this period were not large enough to be resolved by the data. This is in agreement with geological and geochemical observations, which suggest that there have not been major changes in the temporal evolution of the volcano since the onset of gradually increasing fumarollic and seismic unrest in 1998. We expect however that if repeated measurements were done at a higher spatial resolution, for example in the Cratère Sud region where new fumaroles continue to emerge, large conductivity changes would be detected associated to this activity.

Supplementary Fig. S8 represents pseudo-sections of the data acquired using two-dimensional protocols. Measurements performed with remote electrodes are not straightforward to represent, but we refer the reader to^39^ for a representation of the position and combinations of remote injection electrodes and the measurement electrodes. Three mise à la masse profiles^54^ were measured by introducing one of the injections electrodes in the Tarissan acid pond and with the other injection electrode located at the end of the profile. One profile extends from the summit to the west (June 2006), one extends to the south (October 2007) and two crossed profiles extend one to the east and another one crossing it at the base of the dome (July 2011).

### Modelling mesh and data uncertainties

We constructed the unstructured mesh (See Supplementary Fig. S6) used for both the inverse and forward problems with TetGen^56^. Unstructured grid modeling was used to accurately account for topography and prevent from bias in the inference of the internal structure^29^. To avoid boundary problems, the mesh extends 100,000 m to the sides and in depth. A mesh refinement is applied close to the electrodes for numerical precision^29^ (Supplementary Fig. S6). This refinement is achieved by imposing mesh nodes at the surface location of the electrode and 10 cm below it.

A hole of 10 × 30 × 90 m^3^ was included in the mesh to account for the Tarissan pit. The electrode immersed in the Tarissan acid pond was taken into account in the mesh 1 m below the bottom of this pit. No constraints regarding the acid electrical conductivity or depth were included. Similarly, a 10 m wide and 80 m long, south-north oriented hole was included to model the big aperture formed by the Ty fault that propagates from the Tarissan pit through the northern flank of the volcano (Fig. 1b). The bottom of the hole was fixed at 1370 m and thus has a maximum depth of 68 m at the southern extreme and intersects the topography on the dome’s flank in the northern extreme. In practice, including these apertures did not significantly change the inversion results since they are negligible compared to the depth and size of the conductivity structures needed to explain the data.

We tested the sensitivity of the forward responses to mesh parameters such as maximum volume allowed for tetrahedra, electrode refinement, mesh quality, and density of topography points. The latter is the parameter affecting the predictions the most. For all these parameters, we computed residuals comparing the data predicted for the most refined mesh against the immediate version less refined. A trade-off between a more precise mesh and computing times had to be established. We chose parameters such that the predicted data compared to a more precise mesh had relative residuals of less than 20%. Individual data predictions varying more than 20% were seldom observed between the chosen mesh and a more refined version (whatever parameter was being considered). Since these represent a small percentage of the total amount of measurements, we removed these data points from the data set. A total of 452 problematic data points were removed from the data acquired, leaving 23624 data points to invert.

Data errors used in the inversion were estimated from measurements and from the mesh tests. Measurement estimates come from repeated and, when available, reciprocal measurements (See Supplementary Fig. S7). Mesh tests estimates are given by the relative residual between the predicted data of a homogeneous conductivity model calculated with the mesh used in the inversion and a more refined mesh. We also impose an error floor of 5%, such that data points with unrealistically small error estimates were not over-fitted in the inversion. For each data point we take the maximum value of these 3 error estimates (the error floor, the uncertainty estimate form the measurements, and the modeling error form the mesh tests) as the uncertainty to be used in the inversions.

### Smoothness constrained inversion

The three-dimensional inversion was performed using a deterministic smoothness-constrained least-squares algorithm called E4D^29^. Smoothness constraints ensure that adjacent tetrahedra have similar conductivity values while correcting for the under-determination of the inverse problem^57^. The objective function we minimize is given by


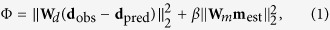


where the subscript 2 denotes the *l*_2_-norm, **d**_obs_ and **d**_pred_ are the observed and predicted electrical potential differences respectively, **W**_*d*_ is the data covariance matrix which we assume to be diagonal, and **W**_*m*_ specifies the smoothness constraints imposed on the model **m**, and **m** contains the logarithm of the electrical conductivity. The first term on the right hand side of Eq. 1 gives a scalar measure of the misfit between the observed and simulated data, the second term without *β* is the corresponding scalar measure of amount of structure of **m**, and *β* is a trade-off parameter that controls the relative contribution of each term.

A starting *β* value is provided which is usually 100 (as in our case). After each iteration *β* is adjusted according to the decrease observed in the objective function: if Φ decreases more than 5%, the value of *β* is kept unchanged, otherwise *β* is reduced to half of its value. Outliers are defined at each iteration as data points whose weighted residuals are larger than 3 times the standard deviation of the weighted residuals’ distribution. These data points are not used for the model update estimation in the next iteration.

### Cross-validation and model uncertainty

Determining an objective trade-off between data fitting and model structure is often complicated, especially so if knowledge of data errors is limited. Here we exploited the large set of data available and performed a cross-validation test^58^. We randomly divided the data in 2 sets and performed separate inversions of each data set. We then compared the RMS


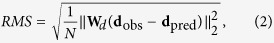


of the inverted data to the RMS of the data that was not used in the inversion (i.e., the validating data set) for each model obtained in the iterative inversion (see Supplementary Fig. S9). As the number of iteration increases, the difference between the validating and inverted data sets’ RMS increases, indicating that the model over-fits the data. We considered the inversion to be over-fitting the data when this difference was larger than 5%. From this threshold, we calculated an optimal RMS for the complete data set (see Supplementary Fig. S9). The model presented in Fig. 2 corresponds to the 16^th^ iteration of the inversion of the complete data set, which is the first model with an RMS value (7.59) similar to the optimal. In practice, model changes are very subtle among iterations at this stage of the inversion. Opting for the next or previous iteration model does not significantly change any of the structures described and interpreted in our work.

Formal uncertainty quantification through a probabilistic approach is in practice not possible due to the the large amount of parameters and the computanional cost of solving the forward problem. In this work we only interpret large-scale features of the model, which are well constrained by our data. All the discussed anomalies have a large influence in the data fit and the RMS would be significantly larger if they were not included in the model. Similarly, we performed tests to evaluate the depth at which the data is not sensitive to conductivity changes and thus avoid misinterpretation of our results (see Supplementary Fig. S10).

## Additional Information

**How to cite this article**: Rosas-Carbajal, M. *et al*. Volcano electrical tomography unveils edifice collapse hazard linked to hydrothermal system structure and dynamics. *Sci. Rep.* **6**, 29899; doi: 10.1038/srep29899 (2016).

## Supplementary Material

Supplementary Information

## Figures and Tables

**Table 1:** Dates and amount of data collected in each acquisition campaign on La Soufrière de Guadeloupe.

Date	Total number of measurements	Percentage of total
April 2003	2208	9.3%
Dec. 2003	3522	14.9%
April 2004	3213	13.6%
May 2005	8302	35.1%
Nov. 2005	1925	8.1%
June 2006	1807	7.6%
Oct. 2007	531	2.2%
Oct. 2010	761	3.2%
July 2011	1355	5.7%
Total	23624	100%
