# Supplementary material for: Volcano electrical tomography unveils edifice collapse hazard linked to hydrothermal system structure and dynamics
Source: Sci Rep. 2016 Jul 26;6:29899. doi: 10.1038/srep29899 (PMC4960541; doi:10.1038/srep29899)
Supplement: Supplementary Information [file srep29899-s1.pdf]

Supplementary Information
Volcano electrical tomography unveils collapse hazard linked to hydrothermal system structure and dynamics

M. Rosas-Carbajal, J-C. Komorowski, F. Nicollin & D. Gibert

1 The La Soufrière of Guadeloupe volcano: brief synthesis of past activity and current unrest

The La Soufrière of Guadeloupe volcano (Fig. 1, S1) belongs to the Lesser Antilles volcanic arc, formed by the subduction of the North American Plate beneath the Caribbean Plate at a rate of ~2 cm.y^{-1}[1]. Over the last 12,000 years its activity has been characterized by a series of dome extrusions, explosive eruptions and partial edifice collapses. At least eight of these collapse events occurred during the last 7,800 years [2, 3, 4].

Catastrophic volcano collapses are often associated with laterally-directed explosive depressurisation (volcanic blasts) of magma and hydrothermal fluids that spread laterally at high speeds (up to 100-235 ms^{-1}) over the volcano flanks causing extensive devastation over tens to hundreds of km^2[5, 6, 7]. About 25 to 60% of the 8 edifice collapses of the last 8500 years that occurred at La Soufrière of Guadeloupe have generated laterally-directed explosions [2, 4]. Recently, laterally-directed explosions on a smaller scale caused 63 fatalities at Ontake volcano in 2014 [8] and could have caused many fatalities at Tongariro in 2012 [9, 10, 11]. Non-magmatic hydrothermal blasts are as mobile as their magmatic counterparts [9, 12].

The La Soufrière lava dome (Fig. S1) was formed during the last major magmatic eruption in 1530 A.D. [13, 3]. A small magmatic explosive eruption was recently discovered by [4] to have occurred on 1657 AD 20 years. Since 1635 AD, six non-magmatic phreatic or hydrothermal eruptions have occurred (about 1 in every 63 years) in different sectors of the dome [2]. The hazards associated with such non-magmatic eruptions include vertical and laterally-directed explosions, ash fall, small-volume pyroclastic density currents, debris flows, acid degassing, and potential contamination of the groundwater. Major non-magmatic eruptions have also produced partial collapses and triggered small laterally-directed hydrothermal explosions associated with significant exurgence of hot acid pressurized hydrothermal fluids (Fig. S2) stored in shallow depth reservoirs within the edifice.

The last eruption in 1976-1977 (Fig. S2) is considered a failed magmatic eruption caused by the intrusion of a small volume of andesitic magma whose ascension stopped about 3 km beneath the surface [14, 15, 16]. The unprecedented intensity of this crisis and the considerable epistemic and aleatory uncertainty as to its evolution towards a paroxysmal explosive eruption similar to the volcanic blast from Montagne Pelée (Martinique) that killed 29,000 people on 8 May 1902 [17], forced the authorities to evacuate 73,000 inhabitants over 6 months. This contested decision, which caused profound socio-economical impact, fostered a major controversy between scientific experts, authorities, the media, and society [2, 18, 19]. Following the 1976-1977 crisis, degassing and thermal flux as well as seismicity reduced gradually to the lowest levels in 1990 since the onset of monitoring in 1950 [2]. Since the end of 1992, a marked increase in the fumarollic degassing flux at the summit of the dome, a progressive increase in shallow seismicity, and a slow rise of temperature of thermal springs close to the dome was detected [20, 16, 21]. In 1998, the sudden onset of high-flux chlorine degassing from

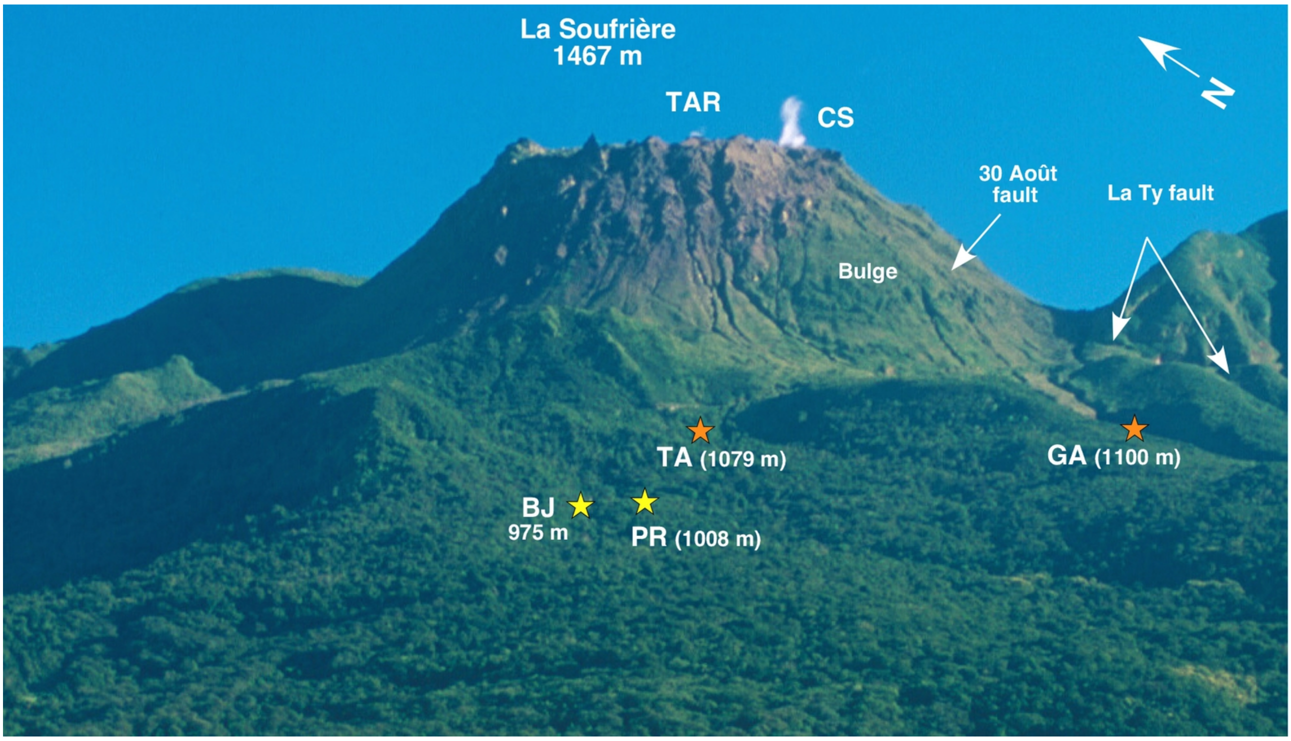

Figure S1: **View of the La Soufrière volcanic massif from the south (1998).** The La Soufrière lava dome was emplaced in 1530 AD within superimposed horseshoe-shaped structures from edifice collapses of the last 3,200 years directed south-southwestward (see Fig. 1b for details). Stars: orange main thermal springs 30-49°C with Galion (GA), Tarade (TA) springs discussed in text; yellow: main thermal springs 24-35°C with Pas du Roy (PR), Bains Jaunes (BJ) springs discussed in text. See Fig. 4b for details and a cross sectional model of the volcano with the position of the conductor regions discussed in the text. In the 1998 photograph, the main vertical gas plume emanates from Cratère Sud (CS). The low flux intermitent degassing that characterized Tarissan crater (TAR) in 1998 has increased recentt years to become permament and the second largest source of gas flux from La Soufrière. The brown area on the dome is the area of vegetation killed subsequently to the onset of high-flux degassing of chlorine-rich hydrothermal and magmatic fluids from Cratère Sud in early 1998. Photo JC Komorowski (IPGP).

the Cratère Sud fumarolle (Fig. S3) marked a significant change in the behaviour of the magmatic-hydrothermal system [22, 2, 15]. Boiling ponds of extremely acid water formed at the Cratère Sud (mean pH between 02-04-1998 to 19-06-2001 of -0.1 with a minimum of -0.8 and maximum of 1.6; Fig. S4) and Tarissan pits (mean pH in 2014 of -0.2 with a minimum of -0.5[23]) in the dome summit in late 1997 and 2001, respectively [22, 2, 15, 23, 20] (Fig. 1). In 2003, the acid pond at the Cratère Sud was replaced by a strongly degassing fumarolle [2]. The Tarissan acid pond continues to be active[23].

The spatio-temporal evolution of surficial manifestations of the hydrothermal system may reflect two main scenarios. Firstly, a reorganization of the pattern of fluid circulation inside the dome might reflect a response to progressive sealing of the formerly active flow paths [36] by hydrothermal alteration. Both the intense hydrothermal activity and the heavy rains (~6-7 $m.yr^{-1}$) supply the hydrothermal reservoirs that favour fluid mineralization by magmatic gas and the formation of clayey material that progressively fills and blocks open fractures in the edifice decreasing its macropermeability[37, 36, 15]. The resulting sealing causes fluid confinement and overpressurization, which eventually lead to the opening of new flow paths inside the edifice. A second possible scenario has been proposed in which the increased flux of summit chlorine-rich degassing and the episodic chlorine spikes recorded in the Carbet and Galion hot springs reflect the sporadic injection of acid chlorine-rich fluids and heat from the magma reservoir or magma intrusions at depth into the hydrothermal reservoirs [38, 39, 15, 16].

2 Historical hydrothermal explosive eruptions at La Soufrière of Guadeloupe volcano

At la Soufrière of Guadeloupe, exegesis and re-analysis of historical chronicles and geological studies have shown [2, 4] that of six historical non-magmatic explosive eruptions that have been documented, the major eruptions of 1797-1798, 1836-1837 and 1976-1977 all have produced laterally-directed explosions and emplacement of associated small volume highly mobile high-energy dilute turbulent pyroclastic density currents that reached probably up to ~1.5-2 km from the volcano [2]. This information is summarized in Fig. S2 and references therein.

Following explosive depressurisation of specific areas of the hydrothermal system through eruptive vents and fractures cross-cutting the dome (Fig. S2), exurgence of pressurised warm to hot acid hydrothermal fluid occurred repeatedly in all of those historical eruptions as well as during the minor 1956 eruption [2]. These observations, coupled with other surface manifestations of the hydrothermal system [2, 15, 16]: fumarollic degassing, thermo-mineral springs, areas of passive degassing, surficial exposures of hydrothermally altered rock (Fig. S5) provide unequivocal evidence of the presence, within the La Soufrière of Guadeloupe volcanic edifice, of numerous possibly interconnected reservoirs of agressive acid hydrothermal fluid contained within otherwise non porous host-rock with low permeabilities. Data from detailed studies of acid hydrothermal systems worldwide (e.g. [39, 40, 41, 42]) show that these fluids promote extensive rock dissolution and alteration. Magmatic or non-magmatic unrest associated with significant increases in heat and gas flux, shallow-depth seismicity, and deformation could trigger physico-chemical instabilities in these reservoirs or their interaction with meteoric recharge water that could promote transient overpressurization of the hydrothermally-altered core of the volcano (Fig. S5) leading to violent phreatic eruptions with associated laterally-directed explosions[43, 8, 9, 2, 11] and potentially threatening the mechanical stability of the edifice to trigger partial edifice collapse that can reach distances of a few kilometers from the dome and be associated with potential devastating mudflows. [44].

These observations from historical eruptions confirm the presence of large volumes of hydrothermal fluids at shallow depth within the edifice and provide ground truth validation of the different conductor regions inferred from three-dimensional imaging of the resistivity structure of the La Soufrière dome and upper hydrothermal system (Fig. S4).

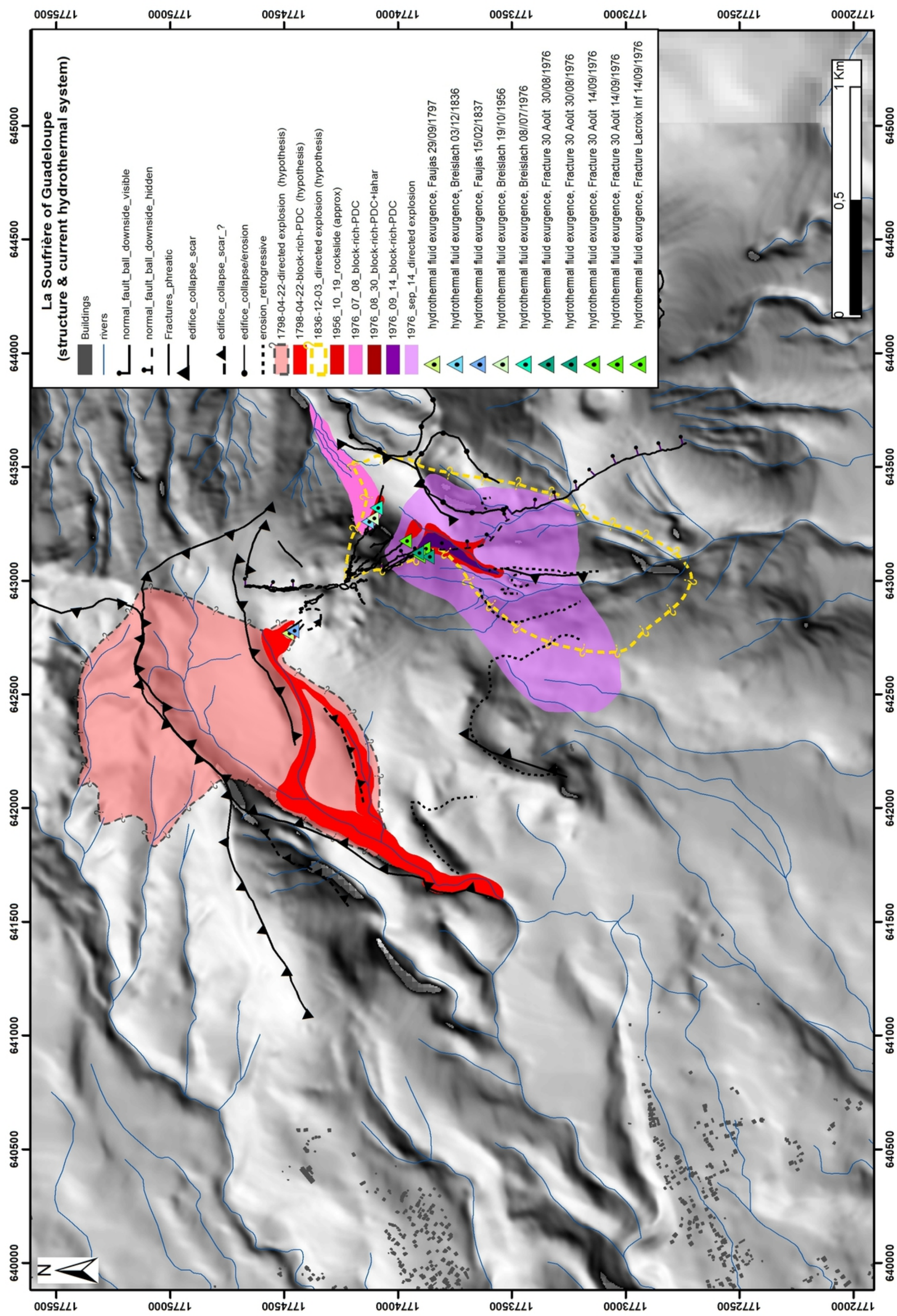

Figure S2: **Reconstruction of phenomenology and deposits associated with historical hydrothermal non-magmatic eruptions at La Soufrière of Guadeloupe between 1798 and 1977.** New data and compilation by J-C Komorowski, 2015 with contributions from Observatoire Volcanologique et Sismologique de Guadeloupe (OVSG) of the Institut de Physique du Globe de Paris (IPGP), modified after [24, 25, 26, 27, 28, 14, 1, 29, 2, 3, 30, 4, 31, 32, 33, 34, 35]. This map was generated using the Esri ArcMapI 10.1 software (http:www.esri.com).

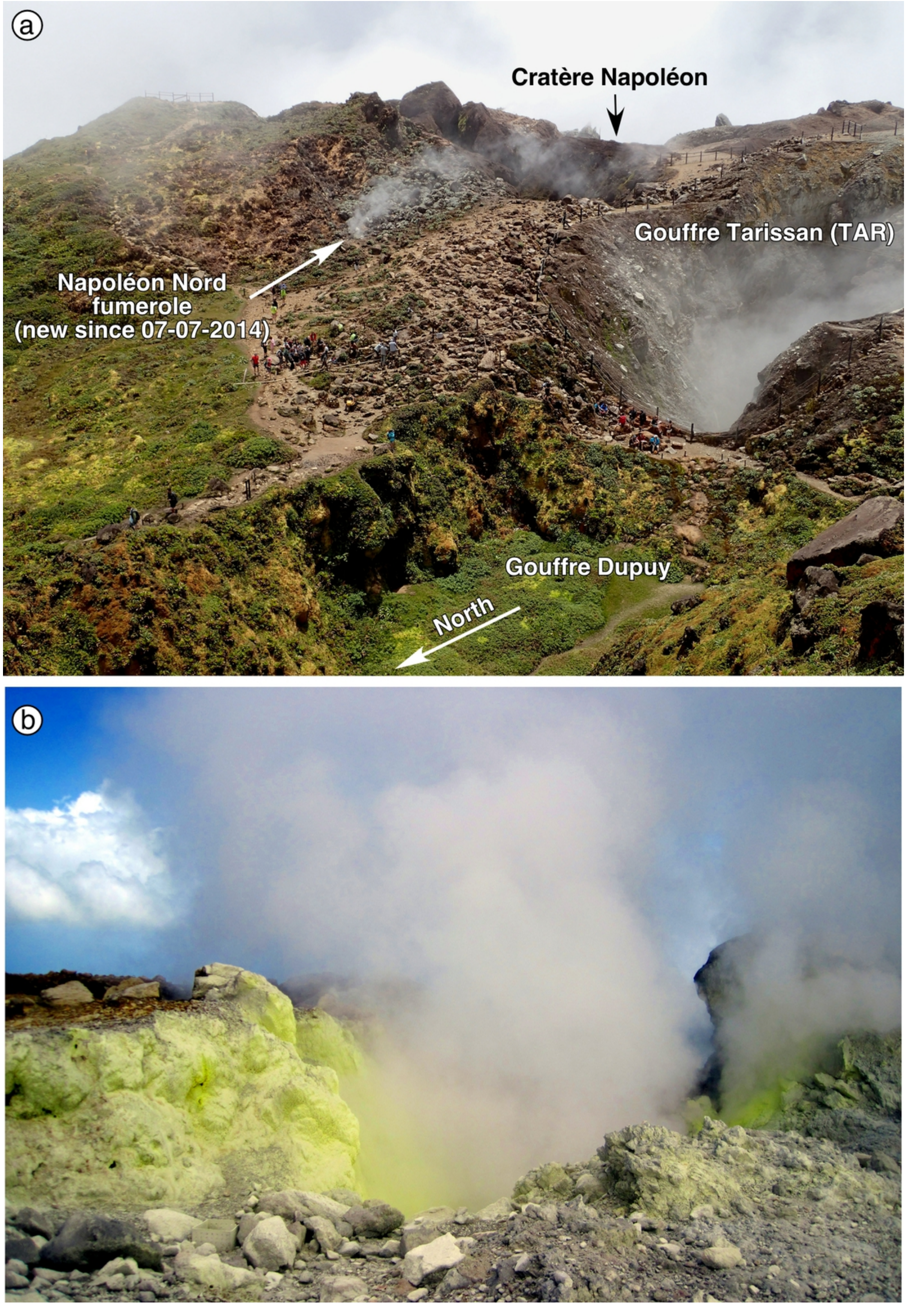

Figure S3: **High flux degassing at the summit of La Soufrière dome.** a) Tarissan and Napoleon North new fumarole. Photo April 2015, JC Komorowski, IPGP. b) Cratère Sud. Photo August 2009, D. Gibert (University of Rennes 1, IPGP).

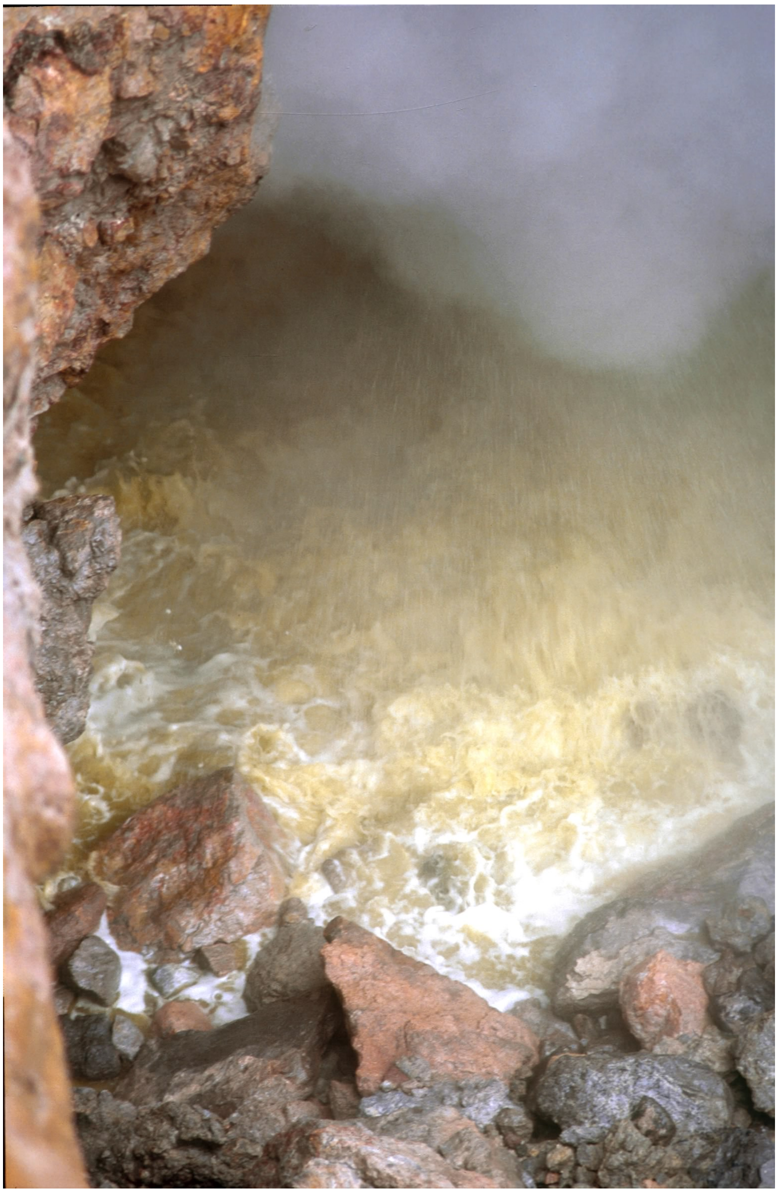

Figure S4: **Hot acid hydrothermal fluids in summit craters at La Soufrière of Guadeloupe volcano.** Cratère Sud (1998) boiling pond with acid (-1<pH<1.5) hydrothermal and magmatic fluids, inferred to be similar in composition to fluids saturating the A2 region. These fluids rise from shallow depth hydrothermal fluid reservoirs within the La Soufrière edifice, and were emitted from eruptive vents during historical eruptions in 1797-1798, 1836-1837, 1956, and 1976-1977. Photo JC Komorowski (IPGP).

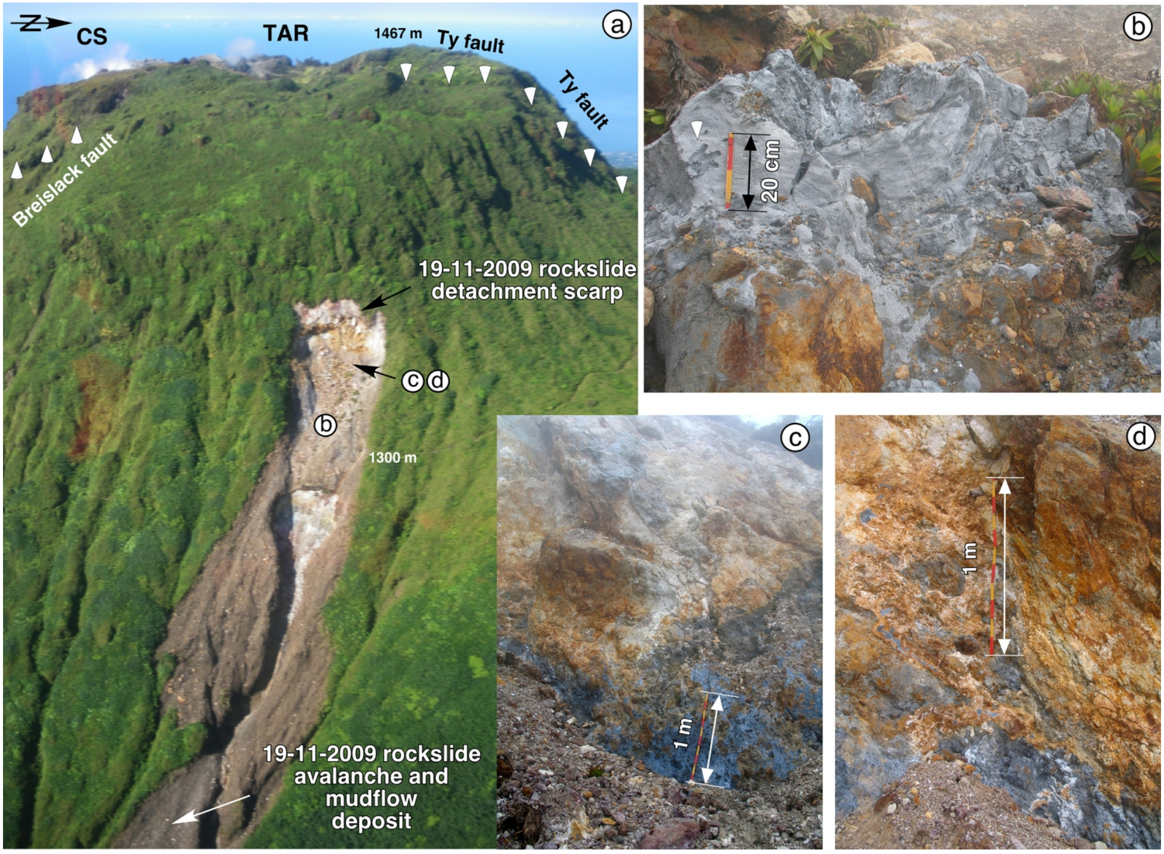

Figure S5: **Superficial rain-triggered rockslide on the La Soufrière dome in 2009 revealing hydrothermally altered core of the dome.** a) Aerial view of the complex collapse scar on the north-eastern flank of La Soufrière (Fig. 1b) that was triggered on 19 November 2009 by one of the most intense rainfall events recorded in 24 hours on La Soufrière in 60 years [20]. The scar is located just above anomaly D (Fig. 1b, 2b). Photo 21-12-2009, JB de Chabalier, IPGP/OVSG. b) Typical grey plastic clay-rich (see imprint of fingers, white arrow) mechanically weak texture of material found inside the dome about 100 m below the summit that constitutes the top of the hydrothermally altered core of the volcano and corresponds to regions of the dome with conductivity values between 0.01 and 0.1 $S.m^{-1}$ (Fig. 2a, 3). This material resulted from prolonged and extensive acid dissolution and alteration of dome rock by rising hydrothermal and magmatic fluids typical of those found in the Cratère Sud acid pond in 1998 (Fig. S4) and at the bottom of the Tarissan pit (Fig. 3). c) The hydrothermally altered core of the edifice is seen at the base of the collapse detachment surface as a grey layer surmounted by moderately altered but fractured coherent dense dome rock that forms the carapace of the dome and that corresponds to the blue region in Fig. 2a, and 4b with conductivity values < 0.01 $S.m^{-1}$. d) Detail of the transition form the altered core to the moderately altered but fractured coherent dense dome rock that forms the carapace of the dome. This recent rockslide provides a valuable control point to derive mechanical rock properties (e.g. density, strength, permeability, porosity) for regions of the dome for which we have obtained and inverted resistivity data. Photos b-d: 15-12-2009, JC Komorowski, IPGP/OVSG.

3 Inverse and forward mesh

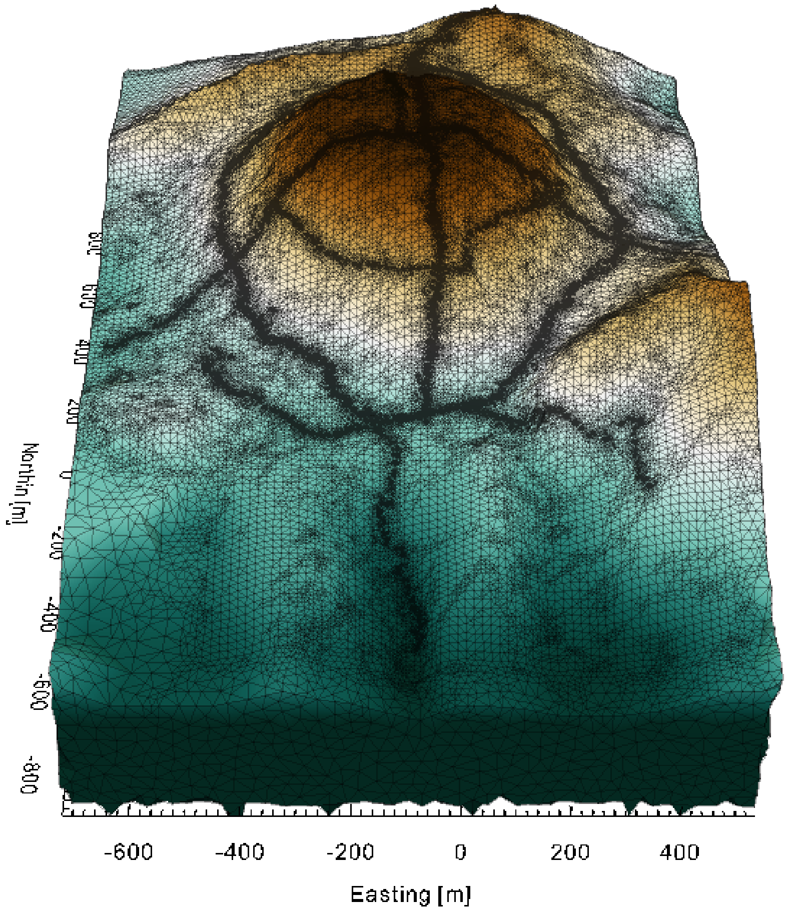

Figure S6: **Central part of the tetrahedral mesh used for the forward and inverse problems.** The dark regions correspond to the mesh refinement around electrodes. To avoid boundary problems, the mesh extends 100,000 m to the sides and in depth (not shown).

4 Measurements and errors

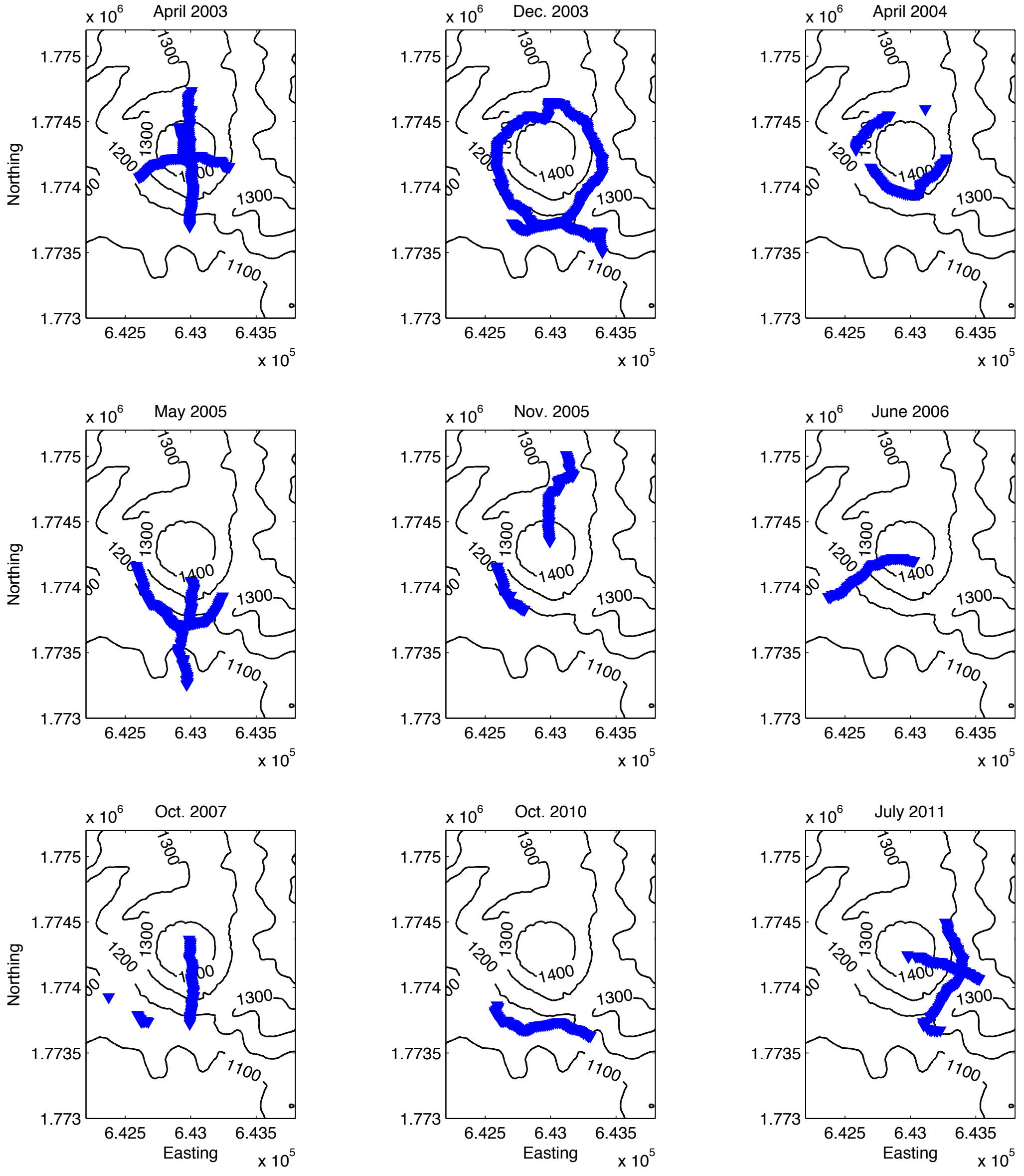

Figure S7: Electrode positions for each data acquisition at different campaigns.

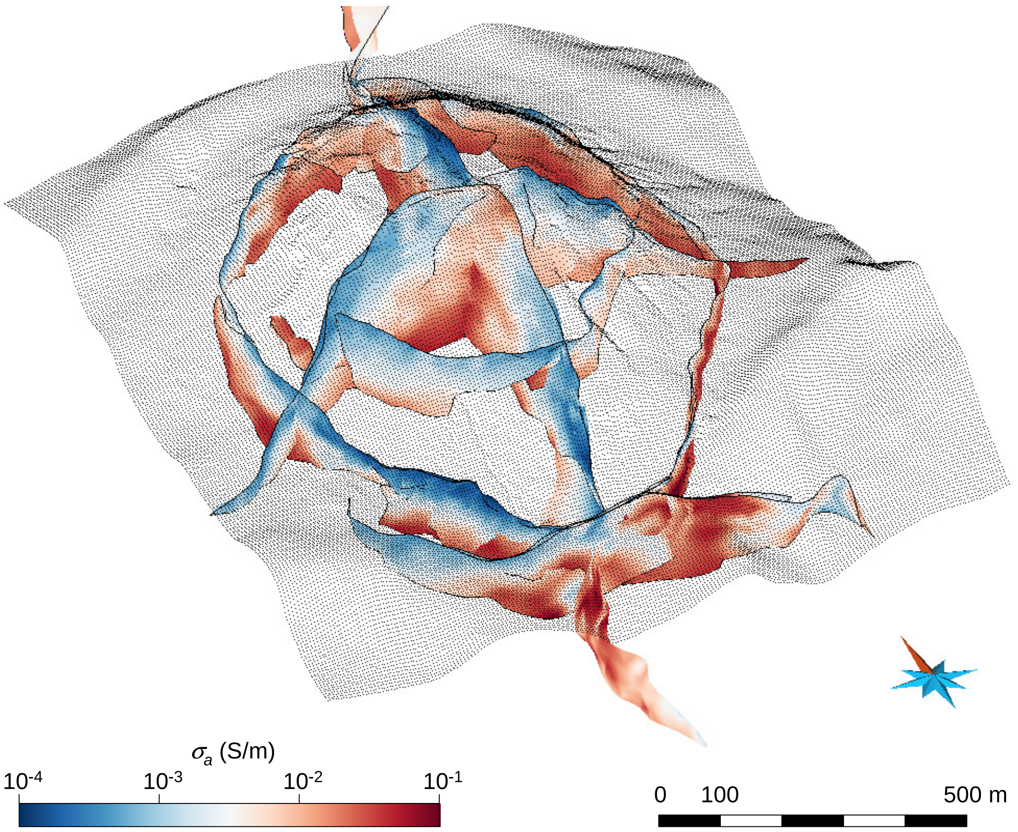

Figure S8: Apparent resistivity pseudo-sections of the data collected using two-dimensional protocols, representing 88 % of the data inverted.

5 Cross-validation of inverted data

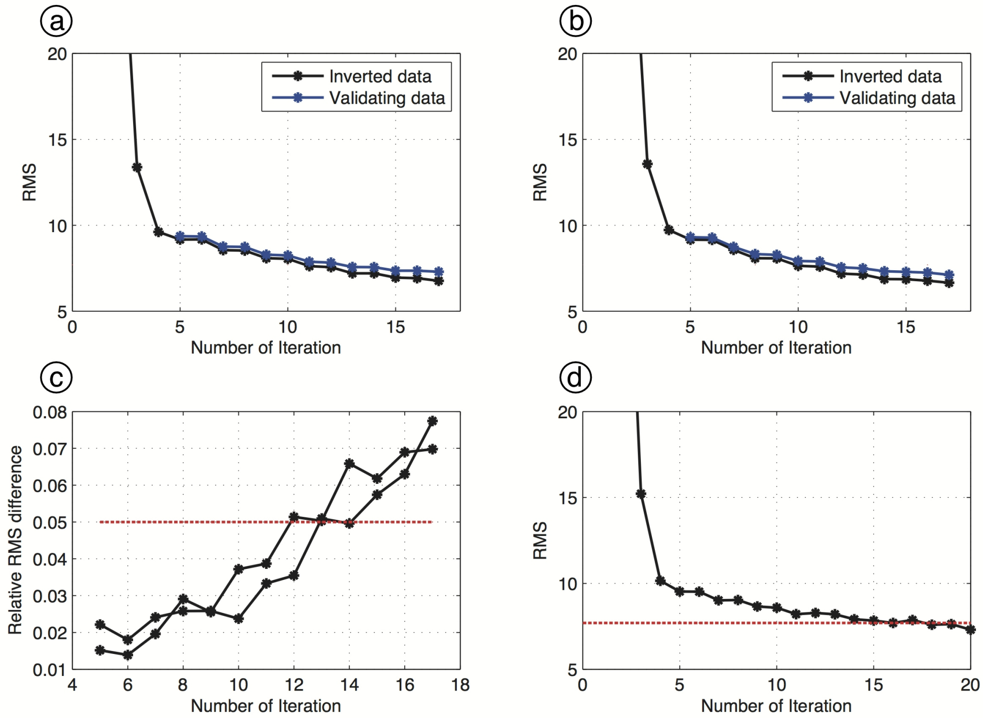

Figure S9: a) and b) Convergence curves and validating RMS of the 2 cross-validation tests done to estimate an appropriate data fit. Data were randomly divided in 2 sets and separately inverted. As the number of iteration increases, the validating data set RMS does not decrease accordingly to the inverted data. This indicates that the model is starting to overfit the data c) Relative RMS difference between the inverted data set and the one used for the cross-validation, for the 2 data sets inverted. The dashed red line indicates a relative difference of 5%. The last iteration to have a difference smaller than this value in both inversions is number 11 (Fig. S9c). The corresponding combined RMSs (considering both the inverted and validated data sets) are 8.0 and 7.6. We thus set a threshold of 7.8 in the inversion of the complete data set. d) Convergence curve corresponding to the inversion of the complete data set. Iteration number 16 (Fig. S9d) provides the first model with an RMS smaller than the threshold (red dashed line), and we therefore considered this model as the final result of the inversion.

6 Data sensitivity to electrical conductivity distribution

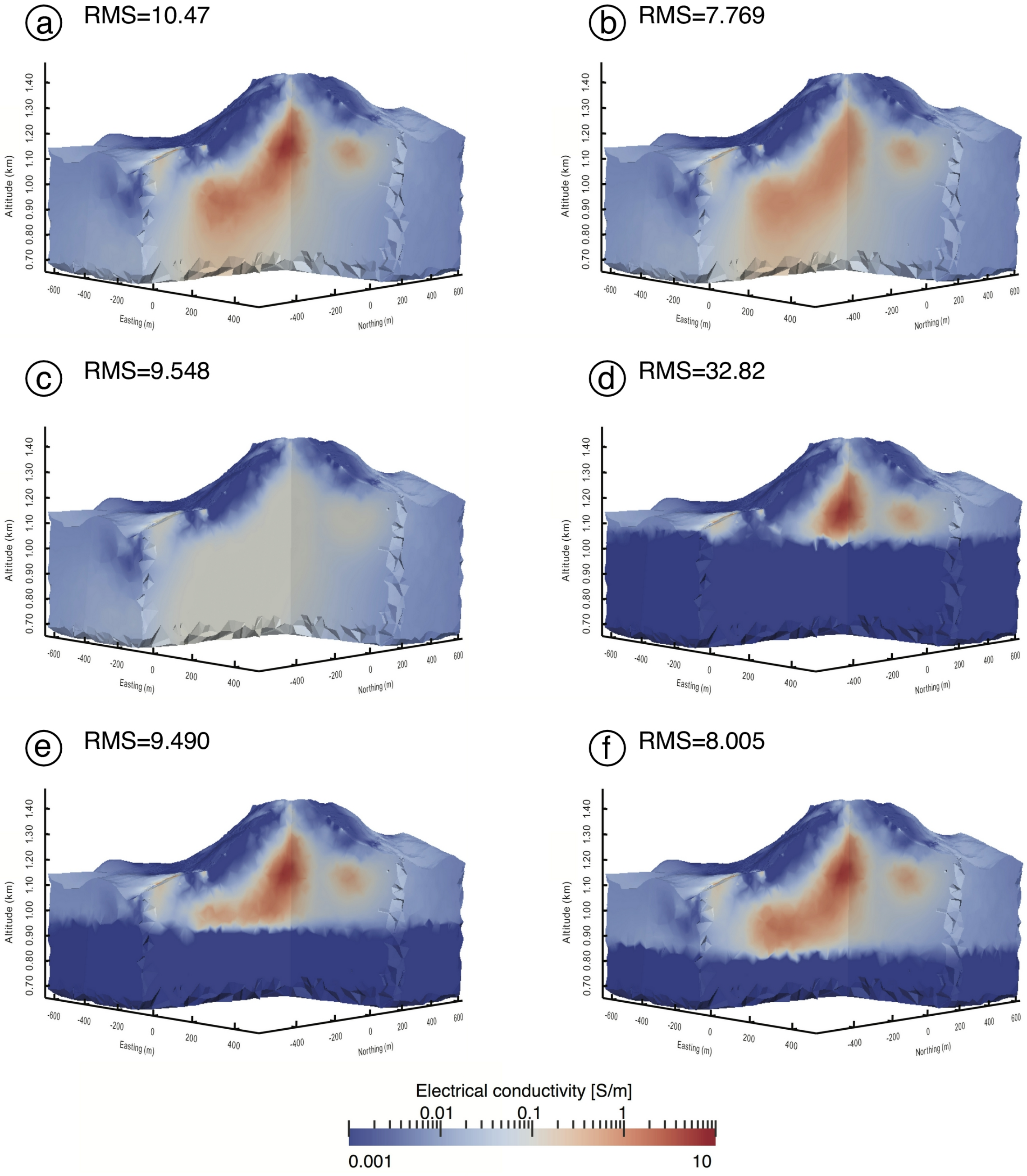

Figure S10: Modified versions of the conductivity model presented in the paper (RMS = 7.59), used to test the sensitivity of the data to the conductivity model obtained. The RMS values correspond to the data misfit of each modified model. a) Conductivity values smaller than 0.001 S/m were replaced by 0.001 S/m. b-c) Conductivities larger than 1 S/m (b) and 0.1 S/m (c) were replaced by each of those values, respectively. d-f) Mesh cells whose center is located below more than 400 m (d), 500 m (e) and 600 m (f) depth from the volcano summit were assigned a conductivity value of 0.001 S/m (the value used for the homogeneous starting model). The data misfits indicate that conductivity values larger than 1 S/m may not be well constrained. Similarly, changing the conductivity values at depths below 600 m from the summit does not significantly affect the RMS. Thus no interpretation should be done about the conductivity structures below 600 m from the summit since the model is not constrained by the data below these depths. Fig. 2 in the manuscript shows the conductivity model until 550 m below the summit.

7 Electrical conductivity model

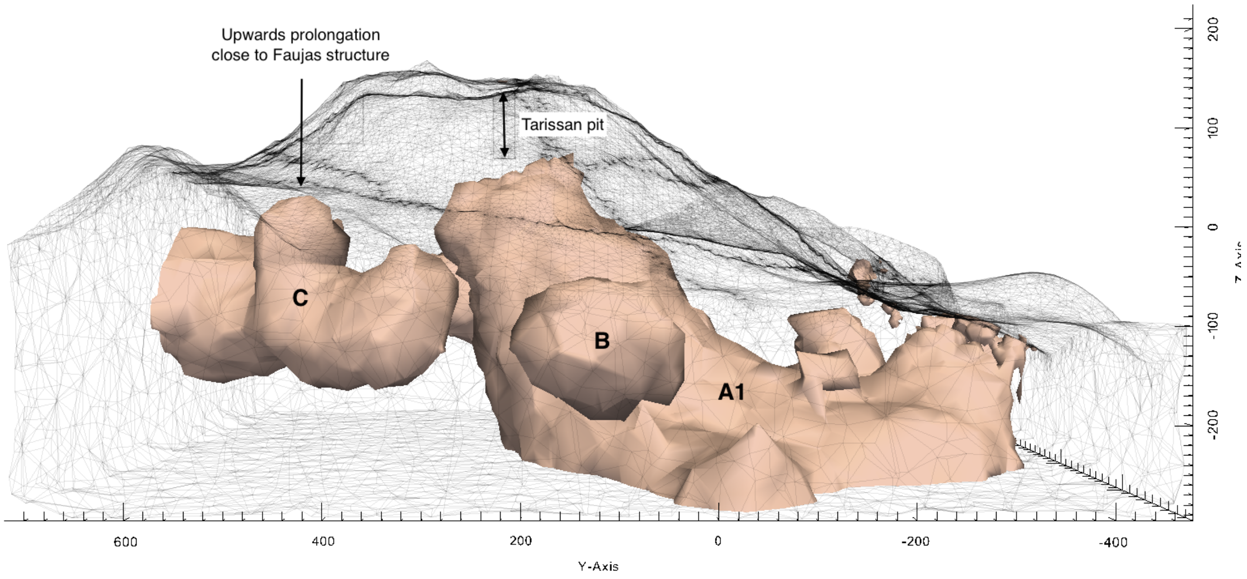

Figure S11: Iso-conductivity surfaces of 0.1 S/m with computing mesh for spatial reference. a,c) View is from west. The depth limit corresponds to the sensitivity limit of the data (Fig. S10)

References

[1] Feuillet, N., Beauducel, F. & Tapponnier, P. Tectonic context of moderate to large historical earthquakes in the lesser antilles and mechanical coupling with volcanoes. *Journal of Geophysical Research: Solid Earth (1978–2012)* **116** (2011).

[2] Komorowski, J. *et al.* Guadeloupe. In *Volcanic Atlas of the Lesser Antilles*, 65–102 (Seismic Research Unit, The University of the West Indies Trinidad, 2005).

[3] Komorowski, J.-C., Legendre, Y., Caron, B. & Boudon, G. Reconstruction and analysis of subplinian tephra dispersal during the 1530 AD Soufrière (Guadeloupe) eruption: Implications for scenario definition and hazards assessment. *Journal of Volcanology and Geothermal Research* **178**, 491–515 (2008).

[4] Legendre, Y. *Reconstruction fine de l'histoire éruptive et scénarii éruptifs à La Soufrière de Guadeloupe: vers un modèle intégré de fonctionnement du volcan. (French) [A high resolution reconstruction of the eruptive past and definition of eruptive scenarii at La Soufrière of Guadeloupe].* Ph.D. thesis, Paris 7 (2012).

[5] Belousov, A., Voight, B. & Belousova, M. Directed blasts and blast-generated pyroclastic density currents: a comparison of the Bezymianny 1956, Mount St Helens 1980, and Soufrière Hills, Montserrat 1997 eruptions and deposits. *Bulletin of Volcanology* **69**, 701–740 (2007).

[6] Sparks, R. *et al.* Generation of a debris avalanche and violent pyroclastic density current on 26 December (Boxing Day) 1997 at Soufriere Hills Volcano, Montserrat. *MEMOIRS-GEOLOGICAL SOCIETY OF LONDON* **21**, 409–434 (2002).

[7] Voight, B. *et al.* The 26 December (Boxing Day) 1997 sector collapse and debris avalanche at Soufriere Hills volcano, Montserrat. *MEMOIRS-GEOLOGICAL SOCIETY OF LONDON* **21**, 363–408 (2002).

[8] Sano, Y. *et al.* Ten-year helium anomaly prior to the 2014 Mt Ontake eruption. *Scientific reports* **5** (2015).

[9] Lube, G. *et al.* Dynamics of surges generated by hydrothermal blasts during the 6 August 2012 Te Maari eruption, Mt. Tongariro, New Zealand. *Journal of Volcanology and Geothermal Research* **286**, 348–366 (2014).

[10] Procter, J. *et al.* Debris flow evolution and the activation of an explosive hydrothermal system; Te Maari, Tongariro, New Zealand. *Journal of Volcanology and Geothermal Research* **286**, 303–316 (2014).

[11] Breard, E., Lube, G., Cronin, S. & Valentine, G. Transport and deposition processes of the hydrothermal blast of the 6 August 2012 Te Maari eruption, Mt. Tongariro. *Bulletin of Volcanology* **77**, 1–18 (2015).

[12] Sekiya, S. & Kikuchi, Y. The eruption of Bandai-san. *Tokyo Imp Univ Coll Sci J* **3:91–172** (1890).

[13] Boudon, G., Komorowski, J.-C., Villemant, B. & Semet, M. P. A new scenario for the last magmatic eruption of La Soufrière of Guadeloupe (Lesser Antilles) in 1530 AD: Evidence from stratigraphy radiocarbon dating and magmatic evolution of erupted products. *Journal of Volcanology and Geothermal Research* **178**, 474–490 (2008).

[14] Feuillard, M. *et al.* The 1975-1977 crisis of La Soufrière de Guadeloupe (FWI): a still-born magmatic eruption. *Journal of Volcanology and Geothermal Research* **16**, 317–334 (1983).

[15] Villemant, B. *et al.* The memory of volcanic waters: shallow magma degassing revealed by halogen monitoring in thermal springs of La Soufrière volcano (Guadeloupe, Lesser Antilles). *Earth and Planetary Science Letters* **237**, 710–728 (2005).

[16] Villemant, B. *et al.* Evidence for a new shallow magma intrusion at La Soufrière of Guadeloupe (Lesser Antilles): insights from long-term geochemical monitoring of halogen-rich hydrothermal fluids. *Journal of Volcanology and Geothermal Research* **285**, 247–277 (2014).

[17] Boudon, G., Le Friant, A., Villemant, B. & Viode, J.-P. Martinique. *Volcanic Atlas of the Lesser Antilles* 126–145 (2005).

[18] Hincks, T. K., Komorowski, J.-C., Sparks, S. R. & Aspinall, W. P. Retrospective analysis of uncertain eruption precursors at La Soufrière volcano, Guadeloupe, 1975–77: volcanic hazard assessment using a bayesian belief network approach. *Journal of Applied Volcanology* **3**, 1–26 (2014).

[19] Komorowski, J., Hincks, T., Sparks, R., Aspinall, W. *et al.* Improving crisis decision-making at times of uncertain volcanic unrest (Guadeloupe, 1976). In *Global Volcanic Hazards and Risk*, 255 (Cambridge University Press, 2015).

[20] OVSG-IPGP. Bulletin mensuel de l'activité volcanique et sismique de Guadeloupe. (French). Monthly public report of Guadeloupe's volcanic and seismic activity (1999-2015).

[21] Allard, P. *et al.* Steam and gas emission rate from La Soufrière volcano, Guadeloupe (Lesser Antilles): implications for the magmatic supply during degassing unrest. *Chemical Geology* **384**, 76–93 (2014).

[22] Komorowski, J. *et al.* L'activité éruptive et non-éruptive de la Soufriére de Guadeloupe: problèmes et implications de la phénoménologie et des signaux actuellement enregistrés. (French). Eruptive and non-eruptive activity from la Soufrière of Guadeloupe: problems and implications posed by the current phenomenology and monitoring signals. Workshop on volcanic hazards - Lesser Antilles volcanoes: from processes to signals. . *PNRN (CNRS)–INSU, BRGM, CEA, CEMAGREF, CNES, IRD* 18–19 (2001).

[23] OVSG-IPGP. Bulletin annuel de l'activité volcanique de la Soufrière de Guadeloupe et de la sismicité régionale. (French). Annual report of La Soufrière of Guadeloupe volcanic activity and regional seismicity (2014).

[24] Ballet, J. *La Guadeloupe: renseignements sur l'histoire, la flore, la faune, la géologie, la minéralogie, l'agriculture, le commerce, l'industrie, la législation, l'administration. (French). Guadeloupe: enquiry on history, flora, wildlife, geology, mineralogy, agriculture, trading, industry, legislation, administration*, vol. 3 (Imprimerie du gouvernement, 1899).

[25] Barrabé, L. & Jolivet, J. Les récentes manifestations d'activité de la Guadeloupe (Petites Antilles). (French). Recent activity manifestations of Guadeloupe (Lesser Antilles) . *Bulletin of Volcanology* **19**, 143–157 (1958).

[26] Biot, M. Daver (1837) La Guadeloupe - Figure explanation, water eruption. *CR Acad Sci Paris IV* 651–654.

[27] Boudon, G., Dagain, J., Semet, M. & Westercamp, D. Carte géologique au 1/20000éme du massif volcanique de La Soufrière. (French). Geological map at 1/20000 scale of the volcanic complex of La Soufrière . Tech. Rep., BRGM-CNRS-DRM-IPGP, Editions BRGM, Orléans, 1 sheet Explanatory notes on the geological map at 1/20000 scale of the volcanic complex of La Soufrière, BRGM-CNRSDRM-IPGP, Ed. BRGM, Orléans, 1-43 (1988).

[28] Brothelande, E. *et al.* Fluid circulation pattern inside La Soufrière volcano (Guadeloupe) inferred from combined electrical resistivity tomography, self-potential, soil temperature and diffuse degassing measurements. *Journal of Volcanology and Geothermal Research* **288**, 105–122 (2014).

[29] Jolivet, J. La crise volcanique de 1956 à la Soufrière de la Guadeloupe. (French). The volcanic crisis of 1956 at La Soufrière of Guadeloupe. In *Annales de Géophysique*, vol. 14, 305 (1958).

[30] Le Guern, F., Bernard, A. & Chevrier, R. Soufrière of Guadeloupe 1976–1977 eruption. Mass and energy transfer and volcanic health hazards. *Bulletin Volcanologique* **43**, 577–593 (1980).

[31] Lherminier, F. Sur les produits du volcan de la Guadeloupe. *Comptes rendus de l'Académie des sciences.* 454–455 (1837).

[32] Lherminier, F. L'éruption du volcan de la Guadeloupe. (French). The volcanic crisis of 1956 at La Soufrière of Guadeloupe . *Nouvelles Annales de Voyage* **74**, 349–350 (1837).

[33] Lherminier, F. Note sur l 'éruption du volcan de la Guadeloupe. (French). On the products from the volcano of La Guadeloupe. *Comptes rendus de l'Académie des sciences.* 294 (1837).

[34] Nicollin, F., Gibert, D., Beauducel, F., Boudon, G. & Komorowski, J.-C. Electrical tomography of La Soufrière of Guadeloupe Volcano: Field experiments, 1D inversion and qualitative interpretation. *Earth and Planetary Science Letters* **244**, 709–724 (2006).

[35] Hapel-Lachênaie, T., Peyre, A. & Fontelliau, C. *Rapport fait aux citoyens Victor Hugues et Lebas, agents particuliers du directoire exécutif aux isles du vent, par la commission établie en vertu de leur arrété du 12 vendémiaire, an 6 de la république, pour examiner la situation du Volcan de*

la Guadeloupe, et les effets de l'éruption qui a eu lieu dans la nuit du 7 au 8 du même mois. (French). Report made to the citizens Victor Hugues et Lebas, special agents of the executive directorship of the Winward Islands, by the commission established on the basis of the official order of the 12 vendémiaire, year 6 of the republic, to examine the situation of the Volcano of La Guadeloupe, and the effects of the eruption that occurred in the night of the 7th to the 8th of the same month (An VI. Facsimile, Société d'Histoire De la Guadeloupe, Basse-Terre, 1798).

[36] Salaün, A., Villemant, B., Gérard, M., Komorowski, J.-C. & Michel, A. Hydrothermal alteration in andesitic volcanoes: trace element redistribution in active and ancient hydrothermal systems of Guadeloupe (Lesser Antilles). *Journal of Geochemical Exploration* **111**, 59–83 (2011).

[37] Zlotnicki, J., Feuillard, M. & Hammouya, G. Water circulations on La Soufrière volcano inferred by self-potential surveys (Guadeloupe, Lesser Antilles). Renew of volcanic activity? *Journal of geomagnetism and geoelectricity* **46**, 797–813 (1994).

[38] Boichu, M., Villemant, B. & Boudon, G. Degassing at La Soufrière de Guadeloupe volcano (Lesser Antilles) since the last eruptive crisis in 1975–77: Result of a shallow magma intrusion? *Journal of Volcanology and Geothermal Research* **203**, 102–112 (2011).

[39] Fournier, R. O. Hydrothermal systems and volcano geochemistry. In *Volcano Deformation*, 323–341 (Springer, 2006).

[40] Giggenbach, W. Redox processes governing the chemistry of fumarolic gas discharges from White Island, New Zealand. *Applied Geochemistry* **2**, 143–161 (1987).

[41] Hochstein, M. P. & Browne, P. R. Surface manifestations of geothermal systems with volcanic heat sources. In *Encyclopedia of volcanoes*, 835–855 (Academic Press, 2000).

[42] López, D. L. & Williams, S. N. Catastrophic volcanic collapse: relation to hydrothermal processes. *Science* **260**, 1794–1796 (1993).

[43] Barberi, F., Bertagnini, A., Landi, P. & Principe, C. A review on phreatic eruptions and their precursors. *Journal of volcanology and geothermal research* **52**, 231–246 (1992).

[44] Reid, M. E. Massive collapse of volcano edifices triggered by hydrothermal pressurization. *Geology* **32**, 373–376 (2004).
